# EEG-based action anticipation in human-robot interaction: a comparative pilot study

**DOI:** 10.3389/fnbot.2024.1491721

**Published:** 2024-12-03

**Authors:** Rodrigo Vieira, Plinio Moreno, Athanasios Vourvopoulos

**Affiliations:** ^1^VisLab, Department of Electrical and Computer Engineering, Institute for Systems and Robotics (ISR-Lisboa), Instituto Superior Técnico, Lisbon, Portugal; ^2^LaSEEB, Department of Bioengineering, Institute for Systems and Robotics (ISR-Lisboa), Instituto Superior Técnico, Lisbon, Portugal

**Keywords:** brain-computer interfaces, human-robot interaction, action anticipation, Convolutional Neural Networks, EEG

## Abstract

As robots become integral to various sectors, improving human-robot collaboration is crucial, particularly in anticipating human actions to enhance safety and efficiency. Electroencephalographic (EEG) signals offer a promising solution, as they can detect brain activity preceding movement by over a second, enabling predictive capabilities in robots. This study explores how EEG can be used for action anticipation in human-robot interaction (HRI), leveraging its high temporal resolution and modern deep learning techniques. We evaluated multiple Deep Learning classification models on a motor imagery (MI) dataset, achieving up to 80.90% accuracy. These results were further validated in a pilot experiment, where actions were accurately predicted several hundred milliseconds before execution. This research demonstrates the potential of combining EEG with deep learning to enhance real-time collaborative tasks, paving the way for safer and more efficient human-robot interactions.

## 1 Introduction

The ubiquity and omnipresence of technology in modern life has raised the necessity in understanding how humans and machines can interact in productive, safe, and fulfilling ways. Specifically, robots represent a new frontier for human-machine interaction, becoming increasingly prevalent and versatile in recent decades. Their capabilities now span a wide range of tasks, significantly impacting various sectors and daily life (Graetz and Michaels, 2018): automated guided vehicles move packages around warehouses (Bogue, 2016); industrial robots assemble complex machinery, such as cars (Brogårdh, 2007); educational robots provide children with an introduction to programming and logic (Atman Uslu et al., 2023); service robots spare users the work of performing tedious household chores (Sahin and Guvenc, 2007); and these are but a few examples from the universe of potential applications. As scenarios of human-robot interaction grow in number, so does the need to address the technical, ethical, economical, and sociological questions behind them. A constant area of research in this field is the search for new ways for humans and robots to collaborate in order to complete a task (Arents et al., 2021), to which the ability to anticipate human action is crucial.

Interestingly, electroencephalography (EEG) can provide us with a way of detecting action intention before its onset. EEG is a functional imaging technique with high temporal resolution, and has been shown to detect changes in brain activity preceding action by over 1-s (Brunia et al., 2011). Further, mobile EEG systems are now more common than ever; they are also relatively inexpensive, non-invasive, and considerably easier to set up than when first introduced (Biasiucci et al., 2019). As a result, EEG signals offer a promising data source for achieving action anticipation in human-robot interaction (HRI) applications.

In addition, modern Deep Learning techniques, such as Convolutional Neural Networks (CNN), can support multi-modal data and end-to-end time series classification, allowing for the extraction of useful action anticipation features from EEG data with very low latency. According to previous works, brain regions responsible for motor action exhibit distinct responses prior to motion onset, which can be exploited to anticipate action through the use of Machine Learning techniques, both during imagined and executed movement (Brunia et al., 2011; Lew et al., 2012; Planelles et al., 2014; Buerkle et al., 2021).

Due to the broad range of contexts in which humans and robots interact, and the different ways in which they do so, it is important to approach any HRI challenge under a clear and concise definition for which interactions we seek to augment. In this work, we will focus on a more strict definition of HRI as the successive manipulation of the same object by a human and a robot. Having established action anticipation as playing a fundamental role in cooperation between humans and robots, the motivation behind a system capable of predicting human limb motion in an accurate, timely manner, becomes clear.

This paper aim to provide a basis for the comparison of Machine Learning methodologies that use EEG signals to anticipate action, by performing an evaluation of different Deep Learning, namely CNN-based, approaches for the task of action anticipation from EEG signals. The resulting action anticipation model should also be capable of anticipating action within a short amount of time. Furthermore, this work aims to compare Deep Learning methodologies for the task of action anticipation using EEG through novel performance metrics, providing the basis for an action anticipation system which can be deployed and evaluated in online HRI experiments.

The main contributions of this work are:

- Proposing and evaluating Deep Learning models for low-latency action detection and anticipation using EEG signals;
- Establishing a novel metric that combines classification time advantage with a measure of its consistency, quantifying each model's action anticipation capabilities;
- Obtaining and analyzing a pilot-experiment dataset for action anticipation in an HRI task, with time-labeled EEG signals.

## 2 Background

### 2.1 Action anticipation in HRI

A wide range of issues must be considered when developing physical robotic systems made to interact with humans. These robots must not only be safe and reliable, but also be perceived as such by their human counterparts. However, the unpredictability of anthropic environments requires robots to behave with at least some degree of autonomy, requiring careful design and control.

In their “Atlas of physical Human-Robot interaction”, De Santis et al. (2008) portray dependability in physical HRI as the interplay between 5 key attributes: safety, reliability, availability, integrity, and maintainability. Of these, safety, in particular, relies on an accurate anticipation and modeling of human action. For physical HRI to be safe, collisions must be avoided, even at the cost of risking task completion. To achieve this, changes to the mechanical design of the robots may be made, such as using lighter materials, or applying compliant transmissions; however, safety can also be improved by equipping the robot or its environment with adequate sensors, with the goal of obtaining a model of human behavior that can inform the robot's control and decision systems.

Robot reliability in physical HRI can also benefit from integration of action anticipation into the control and decision system: more accurate information about human action can improve fault-handling by allowing the robot to identify potential changes in its environment and the objects it is manipulating. By potentially preventing unsafe robot actions, access to this information can also avoid triggering fail-safe mechanisms that lower robot uptime, and may have a downstream negative effect in the production process.

Ultimately, a physical HRI system is made safer and more reliable through the coexistence of proactive and reactive behaviors. To enable the former, action anticipation systems are fundamental.

### 2.2 Electroencephalography for action anticipation

Nearly sixty years ago, Kornhuber and Deecke (1965) published their findings regarding the detection of a readiness potential preceding voluntary, self-paced movement. This *bereitschaftspotential* (BP), referred to using the original German term, is made up of two main components: early BP, beginning approximately 1.5 s before movement onset, has a very low amplitude; late BP starts 0.5 s before action and exhibits greater amplitude, making it easier to detect. This potential can be detected in electrodes placed over the Supplementary Motor Area (SMA) and Primary Motor Area (PMA).

The BP is the earliest of three components which make up Movement-Related Cortical Potentials (MRCPs). As action is executed, a Motor Potential can be detected over the Primary Motor Area, followed by a less pronounced Movement-Monitoring Potential (do Nascimento et al., 2006).

When movement is performed in response to a cue, rather than in a self-paced manner, a Contingent Negative Variation (CNV) occurs rather than the BP. This phenomenon is also a negative potential, but presents a different spatial distribution, originating from a region anterior to that of the BP, the dorsal Premotor Cortex (Pfurtscheller and Silva, 1999). Debate exists about whether late CNV and BP components constitute the same phenomenon, as these exhibit a number of similarities (Grünewald et al., 1979).

Before movement onset, it is also possible to identify Event-Related desynchronization (ERD) starting at roughly the same time as the BP (Pfurtscheller and Silva, 1999), even in people suffering from certain neurological conditions, such as Parkinson's disease (Defebvre et al., 1999). These phenomena consist primarily of contralateral-dominant μ and lower β ERD.

The challenges of action anticipation and the detection of motor execution or motor imagery (MI) using EEG are closely related, though they differ primarily in their application contexts. Motor imagery detection, frequently explored within the scope of restorative BCIs, can typically accommodate a few hundred milliseconds of latency to yield precise detection. The performance of MI-based BCI systems is generally evaluated based on metrics like accuracy and decision rate (often reported per minute). However, in time-sensitive applications, early EEG markers may need to be prioritized over robustness to ensure prompt decision-making. In these cases, system performance is assessed not only in terms of accuracy, but also by the timing advantage it provides in relation to task initiation.

### 2.3 Processing of EEG signals for action anticipation

In the past, action anticipation using EEG signals was generally performed by applying a series of filtering techniques, followed by a linear classifier to detect MRCPs before movement onset (Shakeel et al., 2015). This standard pipeline usually begins with a low-pass FIR filter, followed by spatial filtering, such as through a Large Laplacian Filter (Jochumsen et al., 2013) or an Optimized Spatial Filter (Niazi et al., 2011), and a Matched Filter obtained by averaging training trials to uncover the subject-specific MRCP waveform. Finally, a decision is made by a linear classifier such as SVM (Kato et al., 2011) or LDA (Lew et al., 2012), producing a Receiver Operating Curve which can be used to assess performance characteristics.

This standard pipeline is simple, explainable, and capable of anticipating action with appreciable accuracy and low latency in a laboratory setting. Its simplicity, however, also limits its usefulness in more realistic scenarios: occurrence of Slow Cortical Potentials similar to the MRCP, or movements unrelated to the task at hand lead to a considerable number of false positive detections (Jiang et al., 2015); variations in the MRCP over time due to a number of factors (speed, force of movement, mental effort, subject mood, among others have been identified as altering MRCP characteristics, reviewed in Shibasaki and Hallett (2006) and non-Gaussian noise, such as the result of eye or muscle movement, break the primary assumptions of the Matched Filter (Turin, 1960), degrading system performance.

Recently, developments in Deep Learning have introduced competing strategies for this task: the use of deep Neural Networks, such as Convolutional Neural Networks (Valenti et al., 2021), Long Short-Term Memory Recurrent Neural Networks (Buerkle et al., 2021), and transformers (Al-Quraishi et al., 2022) potentially allows for the detection of these events without resorting to explicit feature selection or extraction, opening the door to end-to-end classification pipelines capable of dealing with the shortcomings of the standard approach.

Another significant trend on this topic is the use of time-frequency decomposition features to detect action-preceding ERD, in conjunction with MRCP, in order to improve robustness (Ibáñez et al., 2014), but calculating these features increases computational complexity and requires the use of larger time windows, potentially introducing latency. Other design choices explored in literature to augment performance include employing more sophisticated spatial filtering techniques (Ahmadian et al., 2013), selecting filter frequency bands in a subject-specific, section wise manner (Jeong et al., 2020), and introducing detection constraints, such as requiring a certain number of consecutive positive classifications, to reduce the amount of false positives and improve consistency (Xu et al., 2014).

### 2.4 State-of-the-art and current gaps

When exploring literature on action anticipation using EEG, it is important to be mindful that the *status quo* on the topic and, more broadly, all of EEG signal analysis, is not well-defined, lacking a consistent, standardized approach to both signal processing and experimental setup (Biasiucci et al., 2019). Nevertheless, identifying gaps requires at least a qualitative understanding of the state-of-the-art solutions proposed.

A major factor to take into account when evaluating the performance of the systems tested is the balance between the True Positive Rate and detection latency, and the False Positive Rate. As the standard pipeline makes use of linear classifiers, designers must make a decision on this trade-off using the convex part of the ROC. Increasing the TPR and detecting movement intention earlier by lowering the detection threshold leads to more spurious detections, which may be unsuited to some applications.

Using a standard pipeline, TPRs in the 70 to 80% range are usually reported, as reviewed in Shakeel et al. (2015). Studies cited in this work present FPRs between 1 and 4 detections per minute, achieving latencies as low as -200 ms. Because most of these studies focus on BCI applications, low FPRs are generally prized over early detections when selecting the decision threshold. Performance generally degrades significantly when the system is tested in an online setting. With Deep Learning methods, Buerkle et al. (2021) achieve accuracies as high as 90%, with considerably earlier detections, anticipating movement by as much as 500 ms in some subjects.

Presently, literature on action anticipation from EEG presents the following gaps: since studies focus on BCI, experiments present low ecological validity for HRI applications, usually consisting of button-pressing or ankle dorsiflexion tasks which are unnatural and unusual in human-robot cooperation environments; exploration of Deep Learning capabilities has been limited; training necessary to develop a Matched Filter is lengthy, making the systems less practical. Additionally, there is no standardized way to represent the reliability/latency trade-off, similar to a Receiver-Operating Characteristic Curve, which could make the integration of these systems in robotic control strategies simpler, by allowing designers of such systems to select the optimal trade-off for specific applications.

## 3 Materials and methods

### 3.1 Experimental stages and setup

The data used to support this work was obtained from two experiments. To ensure model validity, the experimental protocol was similar between experiments. The dataset used to develop and compare tentative models was produced by Farabbi et al. (2022) during a Motor-Imagery BCI experiment with a Baxter Robot (Rethink Robotics, Bochum, Germany). Further, a pilot experiment was conducted, using a Kinova robotic arm Gen3 (Kinova Robotics, Boisbriand, Quebec, Canada) involving realized motor execution, to serve as a proof-of-concept.

During both experiments, participants were seated in front of a robot (a Baxter, in the first stage, and a Kinova Gen3 ultra lightweight robot arm, in the pilot study stage), and had their EEG signals recorded using 32 active electrodes (actiCAP, Brain Products GmbH, Gilching, Germany) through a wireless EEG amplifier (Liveamp 32, Brain Products GmbH, Gilching, Germany) at 500 hertz sampling frequency, including 3-axial accelerometer.

Cues were issued using a 50 Hz LCD monitor placed behind the robot, generated and timed using NeuXus (Legeay et al., 2022), according to the *Graz* paradigm (Pfurtscheller et al., 2003). EEG recordings and cues were synchronized through LabStreamingLayer (Kothe et al., 2014).

Additionally, during the pilot stage, subjects also had their eye movement and pupil data recorded using a Pupil Labs Core headset. Due to data corruption as a result of environmental factors affecting calibration, this modality was not considered for further analysis, but is nevertheless included in the dataset.

### 3.2 Motor-imagery robot-arm control dataset

The dataset used to develop and evaluate the models described in this work was originally produced by Farabbi et al. (2022). The experiment, performed to support the authors' work on Motor-Imagery detection during Robot-Arm Control of a Baxter robot, offers a good starting point for the exploration of EEG-signal classification methodologies during HRI tasks. This context is important, as interaction with robots can have an impact on EEG measurements and classification performance (Rihet et al., 2024).

This experiment involved 12 healthy, BCI-naïve subjects, over the course of three sessions, each with three separate conditions, spanning three phases. The first condition was a resting state, during which the user looked at a neutral visual cue (cross) on a screen in front of them for two minutes. The other two conditions, performed in random order, were first and third-person robot arm control perspectives. Each condition was split into a training and a testing phase. During each of these phases, subjects performed 40 Motor Imagery trials (20 with each arm, in random order), with the start being signalled by a red arrow on screen, during which they were asked to imagine a reaching motion to an object in front of the robot, thus simulating an interaction scenario.

### 3.3 Pilot study

For the pilot experiment, five subjects were recruited, each providing their informed consent. The experiment consisted of two conditions: Motor Imagery (“NOMOVE”), and Motor Execution (“MOVE”), both performed during the same session. During each condition, subjects performed a total of 16 trials, with 8 trials for each arm, in a randomized order. The order in which subjects performed each condition was also random: the initial condition was randomly selected, its trials were performed in succession, and then followed by the other condition's trials.

**Figure 1:**
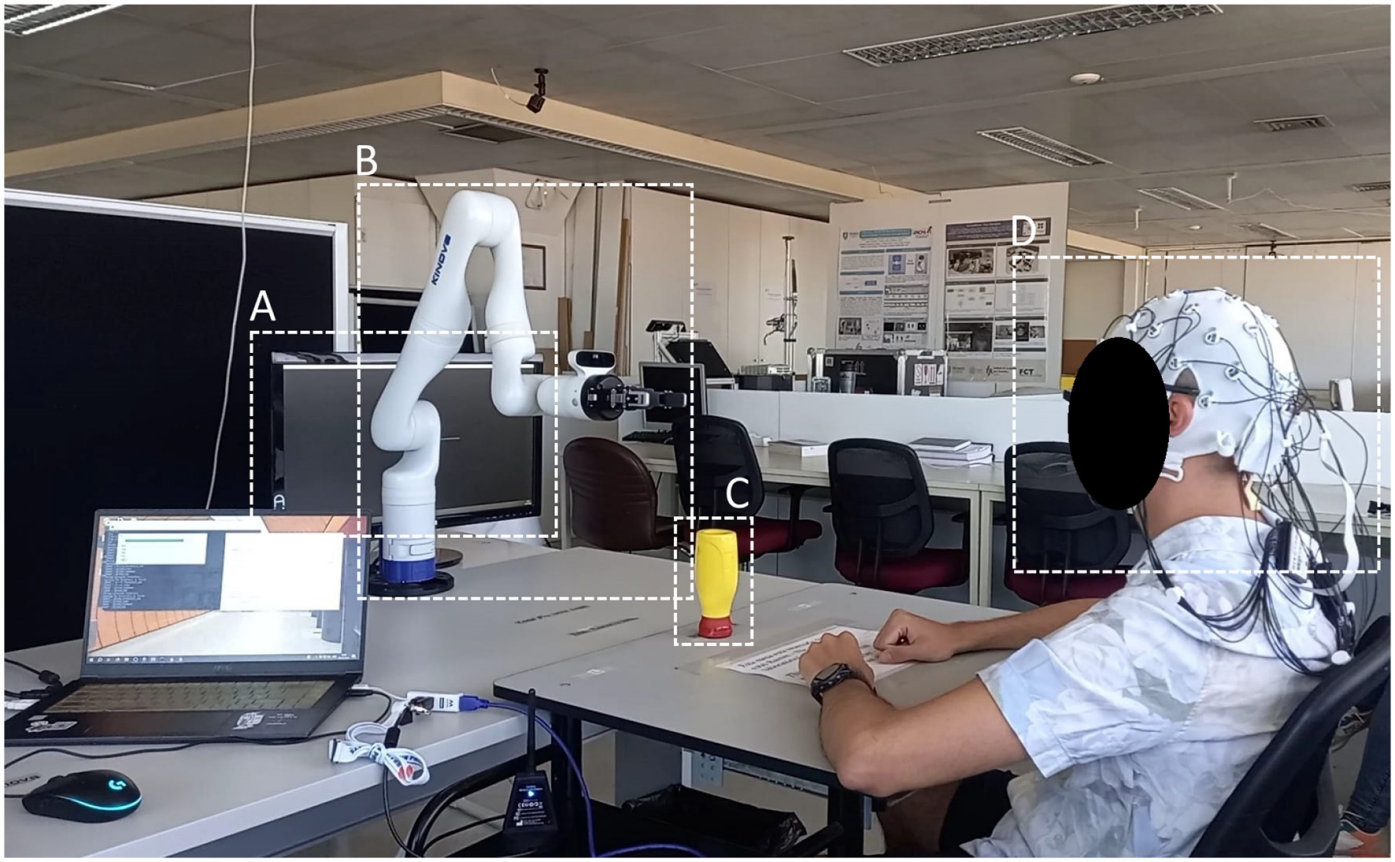
Pilot experiment setup. **(A)** Screen displaying visual cues. **(B)** Kinova lightweight robotic arm. **(C)** Pick-and-place task target object. **(D)** Participant wearing the 32 electrode EEG system.

Participants were sat in front of a robotic arm, behind which was a computer screen displaying “Graz” paradigm cues, and were asked to remain motionless, with each hand placed forward, upon a table in front of them (Figure 1). An object was placed centrally, between the robot and the subject, with markers indicating its 3 possible positions: “L”-left; “C”-central, “R”-right. Before each experiment, a trial run was conducted during which study participants could prepare for the experimental tasks, ensuring they understood the experimental paradigm and could interact safely with the robot.

Experiments began with a 15-s setup wait. During each trial, lasting 18 seconds, a cross would appear on-screen to signal the start for 4 s (baseline); a red arrow would then point to either side of the screen for 1 second; subjects were instructed to, upon disappearance of the arrow, place the object in front of them in the marker corresponding to the side the arrow had pointed, using the arm on that side, during Motor Execution condition, or imagine the aforedescribed movement, during Motor Imagery. The robot would then pick up the object on that side, and place it on its central position once again, coinciding with the end of the trial; during the Motor Imagery condition, the object remained in its central position for the duration of the trials. Trials were followed by an inter-trial period lasting 4 seconds. Each condition lasted for around 8 minutes.

### 3.4 Classification software and hardware

The Deep Learning classification models described, trained, and evaluated in this work were implemented in Python (version 3.11.4) using the *tensorflow* package (v2.12.0) (*keras* backend). Pre-processing was done using the *mne-python* (v1.4.2) and *scipy* (v1.11.4) packages. The machine used to run this software was equipped with an Intel Core i7-8565U CPU (4 cores, 8 logical threads, 1.80 GHz), and 16 GB of RAM, with a Windows 10 64-bit OS.

### 3.5 Signal pre-processing

To prepare EEG data for classification, the following steps were followed: a one-pass, zero-phase, FIR high pass filter was applied at 0.1 hertz to remove baseline drift; a one-pass, zero-phase, FIR notch filter was applied at 50 hertz to remove grid interference; Common Average Referencing was applied, in order to prevent the signal from being biased toward specific areas over the scalp; Independent Component Analysis was then performed on the signal to remove muscle artifact components (Dharmaprani et al., 2016).

The threshold for the removal of ICA components was selected to exclude only blatant artifacts: the goal of this step was not to remove all artifacts, since this cannot be done in an online setting, thus limiting model resilience and validity, but rather to minimize the number of epochs heavily corrupted by artifacts, since the number of examples used to train the models is very low, and this could significantly impact training outcomes.

The signal was then epoched, selecting the intervals spanning [0, 1] (containing action anticipation) and [−1, 0] (no action anticipation) seconds relative to the arrow cue appearing on-screen. Different window lags were also used, in order to characterize action anticipation performance, as will be described in more detail in Section 3.7.

### 3.6 Signal classification models

The following Deep Learning classifiers were evaluated: a Multilayer Perceptron (henceforth MLP); a Deep CNN with an encoding convolutional block and a fully-connected block (CNN); a shallow, fully convolutional CNN (CNN_shallow); and a CNN, Long Short-Term Memory hybrid architecture (CNN_LSTM).

The rationale behind the choice of these architectures was the following: CNNs have been explored in literature for basing numerous BCI systems, as highlighted by Lotte et al. (2018); as such, several CNN-based architectures were evaluated, namely a deep CNN, similar to the one proposed by Cecotti and Graser (2010) for P300 identification, a shallow CNN, which Schirrmeister et al. (2017) demonstrated could outperform FBCSP for MI detection, and a CNN-LSTM hybrid, to explore the viability of combining architectures which had demonstrated the ability to correctly classify action anticipation EEG signals (Buerkle et al., 2021). Furthermore, and considering the low sample size, which can compromise the performance of CNN models, a more generic, rudimentary MLP was implemented, as well as a more traditional Machine Learning approach, using Linear Discriminant Analysis on a Matched Filter output (Niazi et al., 2011).

The MLP classifier was tested to offer a baseline to compare the more sophisticated CNNs against. This classifier features two fully-connected layers with ReLU activation, with *U* units each, and an output unit using sigmoid activation. Use of a dropout layer between each fully-connected layer was also tested.

The CNN classifier features a convolutional block and a fully-connected block. The convolutional block is constituted by a stack of two 2-D convolutional layer and maximum pooling (2 × 2) layer sequences; each convolutional layer features *F* filters, *k*×*k* kernel size, and ReLU activation. The fully-connected block has two fully-connected layers with *U* units and ReLU activation, followed by an output unit with sigmoid activation. Use of a dropout layer between convolutional layers, and between fully-connected layers, was once again tested.

The CNN_shallow classifier is fully-convolutional, based on the shallow architecture featured in the work of Schirrmeister et al. (2017). This classifier features a single convolutional layer, *F* filters, *k*×*k* kernel, followed by a maximum pooling layer (10 × 10), ending with an output unit using sigmoid activation.

The CNN_LSTM classifier is a hybrid architecture, featuring a convolutional encoding block followed by an LSTM decision layer. The convolutional block is similar to the one used for the deep CNN, but is followed by a layer of *U* LSTM units, followed once again by an output unit using sigmoid activation.

Additionally, a more traditional Machine Learning approach was implemented, using an Effect-Matched Spatial Filter to generate a surrogate channel (Schurger et al., 2013), followed by a Matched Filter produced by averaging training trials, with classification performed by Linear Discriminant Analysis. This method has been used extensively for MRCP detection for action anticipation (Niazi et al., 2011).

Alternative approaches considered, implemented, but excluded from this paper include an LSTM DNN, and a Low-Frequency Asynchronous Detector-based classifier. The former was excluded due to poor performance relative to training time, and the latter is not shown as it achieved (marginally) lower performance than the MF approach, which was thus selected to represent more traditional methodologies. Time-frequency decomposition prior to classification was also explored, using Short-Time Fourier Transform, and Gabor Wavelets, both of which underperformed relative to temporal data classification.

### 3.7 Time advantage estimation

The task of action anticipation seeks to provide a robotic control and decision system with a time advantage relative to the onset of human motion. However, in the literature, examples of a unifying time advantage metric are scarce, and generally context-specific, as previously highlighted. Let the Total Time Advantage (TTA) the action anticipation system provides, relative to movement onset, be expressed as the difference between the computational time the system requires to output a decision (henceforth Computational Time Delay (CTD)), and the time advantage of the earliest sample the classifier consistently labels as preceding action, which we will refer to as Decision Time Advantage (DTA).

Obtaining a measure of CTD is straightforward: the computation time spent applying pre-processing and making a prediction for each epoch must be tallied, and a distribution may then be estimated. The computational time delay is the sum of the processing and inference time spent per epoch.

Defining the DTA requires handling the trade-off between decision reliability and latency. Due to false positives, taking the earliest anticipation-labeled sample would provide an unrealistic measure; conversely, requiring the classifier to make an uninterrupted string of positive classifications could provide an overly pessimist reading, and put an arbitrary restriction on downstream applications, which may prize low latency over precision. Nevertheless, the balance between spurious detections and time advantage provided is generally opaque.

We may thus combine latency measures with a measure of classification consistency to produce our DTA. As such, we will define our DTA based on a consistency parameter *k*, such that our metric DTA_*k*_ becomes: the earliest point in time at which the classifier reaches a true-positive rate of *k%*, and after which this value remains above *k*.

To estimate this value, a sliding window was moved from 1 second before the cue to cue onset, in 0.1 second time increments; all pre-processing steps were applied to samples generated this way, labeled by the classifiers. The accuracy at each of these time increments was computed, and PCHIP interpolation was performed to produce a spline which could be used to obtain an estimate of DTA_*k*_ for each classifier at several *k* values. Note that, since positive labels were considered true positives up to 1.5 seconds before movement onset (when MRCP is expected to begin), the accuracy and TPR are effectively the same for the sliding windows used.

### 3.8 Training and performance evaluation methodology

The *Adam* optimizer (Kingma and Ba, 2017) was used to train the classifiers, with a binary cross-entropy loss. Classifiers were trained for each subject/condition/session trio. The trained models were saved, and then evaluated on a validation set, randomly sampled from all epochs (20% validation split). Model performance was evaluated based on four different metrics: accuracy, precision, recall, and F1-score (harmonic mean of precision and recall). For each model parameter, a performance analysis was conducted, in order to select the most adequate values. Final parameters and network configurations are presented in Tables 1–4.

**Table 1:** MLP model parameters (naming according to keras layer used).

**Layer**	**# Filters/units**	**Filter size**	**Stride**	**Activation**
Input	-	-	-	-
Dense	128	-	-	ReLU
Dense	128	-	-	ReLU
Dense	1	-	-	Sigmoid

**Table 2:** CNN model parameters (naming according to keras layer used).

**Layer**	**# Filters/units**	**Filter size**	**Stride**	**Activation**
Input	-	-	-	-
Conv2D	16	9 × 9	1 x 1	ReLU
MaxPooling2D	-	2 × 2	1 x 1	-
Conv2D	16	9 x 9	1 × 1	ReLU
MaxPooling2D	-	2 × 2	1 x 1	-
Flatten	-	-	-	-
Dense	64	-	-	ReLU
Dense	64	-	-	ReLU
Dense	1	-	-	Sigmoid

**Table 3:** CNN_shallow model parameters (naming according to keras layer used) using time-frequency features.

**Layer**	**# Filters/units**	**Filter size**	**Stride**	**Activation**
Input	-	-	-	-
Conv2D	8	2 × 2	1 × 1	ReLU
MaxPooling2D	-	10 × 10	1 x 1	-
Flatten	-	-	-	-
Dense	1	-	-	Sigmoid

**Table 4:** CNN_LSTM model parameters (naming according to keras layer used).

**Layer**	**# Filters/units**	**Filter size**	**Stride**	**Activation**
Input	-	-	-	-
Conv2D	8	2 x 2	1 x 1	ReLU
MaxPooling2D	-	2 x 2	1 x 1	-
Conv2D	1	2 x 2	1 x 1	ReLU
MaxPooling2D	-	2 x 2	1 x 1	-
LSTM	8	-	-	ReLU
Dense	1	-	-	Sigmoid

From the first stage dataset, data were used from all 12 subjects, over the three sessions, in the training (offline) phase of the first and third person conditions, resulting in 72 datasets. Since the pilot dataset features only 5 subjects, performing two conditions over a single session, 10 train/validation runs were performed to obtain a more representative performance distribution.

### 3.9 Statistical analysis

To compare the accuracy of each different classifier, the Mann-Whitney U test was employed (Singh et al., 2015). Mann-Whitney U is a non-parametric test of the hypothesis that, given two samples *x* and *y* from different populations, the probability that *x* is greater than *y* is equal to the probability that *y* is greater than *x*. This method was chosen due to the low sample size and non-normal distribution of the populations. A significance level of 5% was admitted.

## 4 Results

### 4.1 Classifier architecture performance

**Figure 2:**
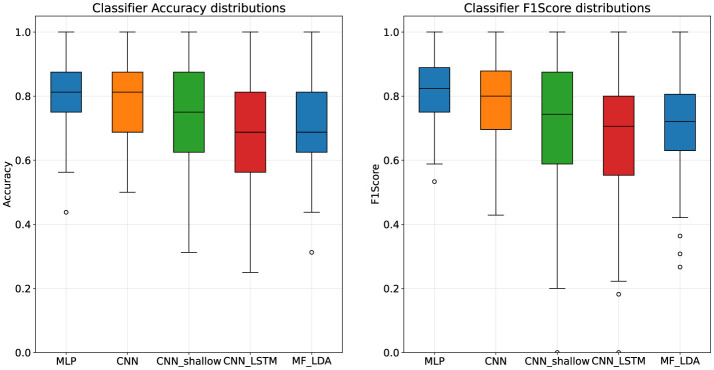
Classifier architecture performance distributions, first stage dataset.

Among the four models tested, the MLP classifier presented the highest classification scores, with a mean accuracy of 80.9% and F1-Score of 81.54%, followed by the deep CNN, presented in Table 5. According to results the of the Mann-Whitney U test, MLP significantly outperforms the CNN_shallow (*p* = 0.012) and CNN_LSTM (*p* < 0.001) classifiers, but not the deep CNN (*p* = 0.317). Furthermore, a look at the distribution of the results reveals very wide intervals for the CNN_shallow and CNN_LSTM classifiers, as visible in Figure 2, with very considerable differences between subjects.

**Table 5:** Classifier architecture performance, first stage dataset.

	**Accuracy**		**F1-score**		**Precision**		**Recall**	
**Classifier**	**Mean**	σ	**Mean**	σ	**Mean**	σ	**Mean**	σ
MLP	80.90	13.05	80.62	13.74	83.51	13.15	81.54	12.08
CNN	78.73	16.86	81.15	14.72	76.04	15.83	77.88	13.88
CNN_shallow	73.87	16.28	75.88	20.57	69.27	22.91	71.22	20.64
CNN_LSTM	68.40	16.86	67.73	25.16	64.41	26.41	64.50	24.36
MF-LDA	71.35	15.51	71.27	15.92	73.01	17.96	72.05	18.81

### 4.2 Classifier decision time advantage

**Figure 3:**
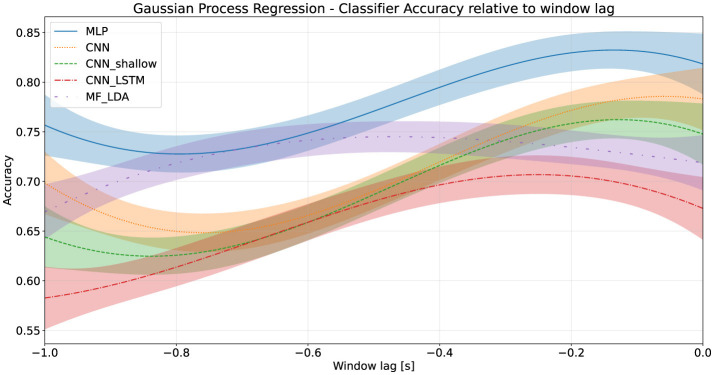
Classifier accuracy relative to training window lag, first stage dataset.

Each classifier was evaluated on a moving time window to determine its action anticipation performance. All classifiers experienced a significant (*p* < 0.001 of first coefficient in linear regression t-test) drop in performance as the window moved earlier. The Decision Time Advantage was estimated at several thresholds *k* using a Gaussian Process Regression. Using a threshold of 75%, the MLP classifier provides a DTA of around 590 milliseconds (95% CI: 520–870 ms), while the DTA_75_ of the deep CNN was lower, at approximately 290 milliseconds (95% CI: 210–360 ms). These results are presented in Table 6 for several thresholds, as well as graphically in Figure 3.

**Table 6:** Classifier decision time advantages for thresholds 80%, 75%, 70%, first stage dataset.

**Classifier**	**DTA_80_ (ms)**	**DTA_75_ (ms)**	**DTA_70_ (ms)**
MLP	380 [290–450]	590 [520–870]	>1,000 [790–1,000]
CNN	–	290 [210–360]	470 [410–530]
CNN_shallow	–	–	460 [390–510]
CNN_LSTM	–	–	–
MF-LDA	–	–	890 [810–980]

95% confidence intervals between square brackets.

The delay introduced by the computational burden of classifying each epoch was also evaluated. This Computational Time Delay can be broken down into time spent with data preprocessing, which is similar for all classifiers, and inference time, largely dependent on classifier architecture. The preprocessing time spent for each epoch was on average 14 ms (μ: 13.97 ms, σ: 5.10 ms). Mean inference times varied between 174 ms, for the shallow CNN, and 799 ms, for the CNN-LSTM hybrid. The total Computational Time Delay for each classifier is presented in Table 7.

**Table 7:** Classifier computational time delay (preprocessing and inference), first stage dataset.

**Classifier**	**Mean (ms)**	**σ (ms)**	**Relative to lowest**
MLP	422.6	431.9	2.27
CNN	270.1	651.3	1.45
CNN_shallow	186.5	341.8	1
CNN_LSTM	814.0	646.3	4.36

### 4.3 Pilot experiment results

**Figure 4:**
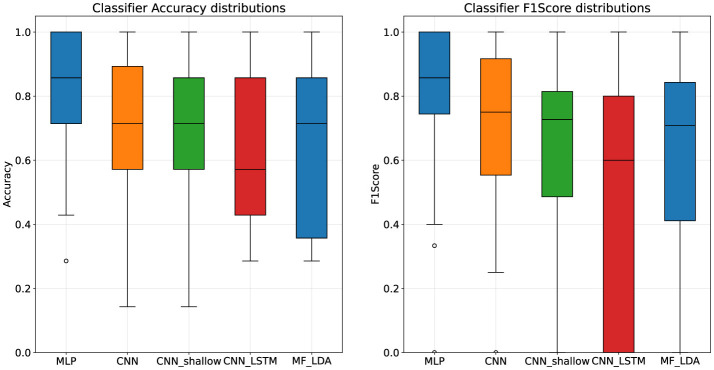
Classifier architecture performance distributions, pilot stage dataset.

When evaluated on the pilot experiment dataset, the MLP remained the most accurate classifier, with a mean accuracy of 82.29%, and F1-Score of 80.63%. Deep CNN classification dropped considerably, with an accuracy of 71.57%. Results are presented in Table 8, Figure 4.

**Table 8:** Classifier architecture performance, pilot stage dataset.

	**Accuracy**		**F1-score**		**Precision**		**Recall**	
**Classifier**	**Mean**	σ	**Mean**	σ	**Mean**	σ	**Mean**	σ
MLP	82.29	17.74	80.63	22.90	82.15	24.28	81.42	24.69
CNN	71.57	23.43	69.20	29.18	70.51	31.22	72.00	31.57
CNN_shallow	68.43	20.23	62.08	31.50	66.32	34.72	64.67	36.33
CNN_LSTM	63.86	19.53	51.64	36.50	51.38	38.29	58.33	42.59
MF-LDA	64.29	25.75	57.75	33.74	56.00	33.98	63.33	37.86

**Figure 5:**
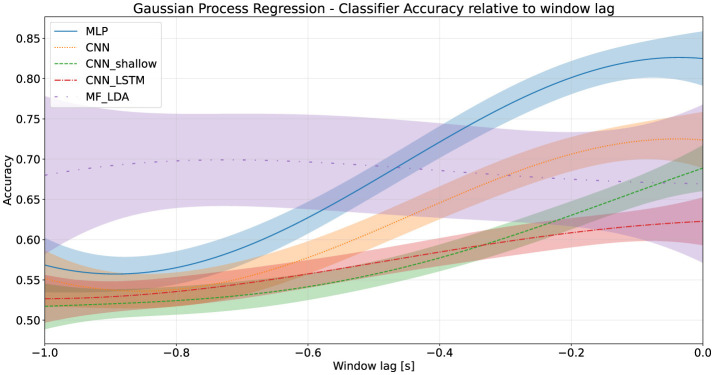
Classifier accuracy relative to training window lag, pilot stage dataset.

The Decision Time Advantage provided by the classifiers at all thresholds was also appreciably worse: at a 70% accuracy threshold, the MLP yielded an advantage of approximately 477 milliseconds, followed by the deep CNN at 256 milliseconds. Values at other thresholds are shown in Table 9, and in Figure 5. Performance deterioration as a result of moving the signal time window was considerably more pronounced during the pilot experiment when compared to the first stage dataset, for both the best performing classifiers, MLP and the deep CNN.

**Table 9:** Classifier decision time advantages for thresholds 80%, 75%, 70%, pilot stage dataset.

**Classifier**	**DTA_80_ (ms)**	**DTA_75_ (ms)**	**DTA_70_ (ms)**
MLP	200 [110–260]	340 [290–380]	440 [400–480]
CNN	–	–	230 [70–300]
CNN_shallow	–	–	–
CNN_LSTM	–	–	–
MF-LDA	–	–	–

95% confidence intervals between square brackets.

## 5 Discussion

### 5.1 Classifier architecture performance

An analysis of the performance of the different classifier architectures reveals two clear stand-outs: the MLP and the deep CNN. Considering MLPs are a rudimentary architecture, generally outperformed by more sophisticated models in most applications, this result is striking. Several factors may have led to the superiority of the MLP: first and foremost, the extremely low number of training examples does not favor the convolutional architectures, usually trained on several thousand data points; second, the MLP constitutes a far more generic architecture, with better classification performance suggesting it could be better suited to detection the time-domain patterns that reveal action anticipation and execution, namely MRCPs than convolution-based models.

### 5.2 Classifier decision time advantage

Through the use of the proposed Decision Time Advantage metric, determining a classifier's action anticipation performance and how it behaves over time becomes more transparent. This methodology is simple enough to be applied to other action anticipation pipelines, even those not based on EEG signal classification. With a quick look at a graphical representation of performance, a robotic control system designer can determine the adequate anticipation-reliability trade-off for a specific application.

The MLP and deep CNN classifiers tested, using a 75% threshold, were able to issue an accurate action anticipation classification with a 590, and 290 millisecond time advantage, respectively. In a high-performance human-robot cooperation scenario, this advantage could prove vital for a timely reaction to human movement, potentially preventing accidents and increasing the speed at which tasks may be performed.

It is important to note, however, that the proposed approach aims only to distinguish between idle and anticipation/action periods; for a more effective interaction, the system would not only need to perform this binary classification, but also decode the movement which is about to take place. Such an extension of this methodology could credibly be implemented, as evidenced by motor cortex functional mapping studies, but could impact the Decision Time Advantage.

To evaluate the Total Time Advantage provided by the system with each of these classifiers, one can subtract the mean Computational Time Delay, shown in Table 6 (first stage) and Table 9 (pilot stage), from the Decision Time Advantage to get a (hardware-specific) estimate. This way, the MLP nets a TTA of roughly 170 milliseconds, while the CNN achieves 20 milliseconds of anticipation, if we consider the DTA_75_, with the hardware used.

### 5.3 Comparison with other state-of-the-art

Ehrlich et al. (2023) explore the potential of approaches based on anticipatory brain signals for Human-Robot Collaboration, sharing our motivation. In their study, human participants collaborate with a robotic partner to follow a pre-defined path, through a computer keyboard. Along this path, certain positions would trigger a takeover situation: a switch between the robot acting autonomously, and human control. The goal of their analysis was to correctly anticipate these takeover situations, through the use of a regularized LDA classifier on ERP features. Accuracy results were generally in line with chance-level, only exceeding these values when discriminating between human takeover versus robotic continuation, at 57.9 ± 4.3%. While a direct comparison cannot be made between the results achieved by this group and ours, namely due to the significantly different interaction paradigm, as well as the lack of a time advantage estimate, the similarity in motivation—using EEG to augment HRI—makes this work notable.

Adjacent to the challenge of anticipation action intention is the problem of discriminating between which action is about to be taken, as tackled by Duan et al. (2021). This group makes use of Task-Related Component Analysis to optimize MRCP data, which is then provided to an LDA for classification. Accuracies as high as 90.01 ± 9.97 % are reported when discriminating between elbow flexion and rest periods; for different actions, results ranged from 82.14% (pronation) to 90.32% (elbow extension) on average. While these results exceed those achieved in our analysis, it should be noted that the tasks performed had a shorter duration, and did not involve interaction, resulting in different MRCP modulation, both in terms of amplitude and latency, which influences results. Furthermore, an estimate of the time advantage achieved is also not provided.

Kostiukevych et al. (2021) also explore the use of CNNs, as well as RNNs, for the anticipation of grasp-and-lift tasks, using the large GAL dataset (Luciw et al., 2014). The Deep Learning models were employed in an end-to-end manner, without previous feature extraction, to analyse the feasibility of different configurations. The AUC of each classifier was plotted relative to the number of time samples used, i.e. the window length. The best performance was achieved by LeNet (LeCun, 2015), reaching AUC of 0.90 in some cases, when using 1000 sample-long windows. Strikingly, much as in our analysis, the hybrid CNN-LSTM tested offered unexpectedly poor performance.

Finally, Buerkle et al. (2021) leverage an LSTM-RNN for action anticipation in a simulated Human-Robot Collaboration environment. Their system, which also performs end-to-end classification of intention vs anticipation epochs, is capable of providing a time advantage between 54 and 513 milliseconds, with reported accuracy between 84.98 and 92.08% (although it is unclear whether they use balanced accuracy). These results are in line with those achieved during our analysis, and further prove the feasibility of the EEG-based approaches to action anticipation.

Overall, the results found in recent literature for analogous experimental paradigms are similar to those achieved by our system. Additionally, the consistent lack of reporting on time advantage provided by other proposals, as well as its ambiguity, when it is reported, solidifies our case for the need for a metric that clarifies not only the time advantage a system can provide, but its trade-off with system reliability.

### 5.4 Pilot experiment

When comparing the results achieved during the first stage and pilot experiment dataset, it is important to be mindful of the impact of the differences between each setup. Most notably, the inclusion of a Motor Execution condition during the pilot experiment likely produces more pronounced movement artifacts, which corrupt the EEG signal. While action anticipating brain potentials such as the BP present low amplitudes, and are often difficult to identify during signal trial analysis, movement artifacts often exceed the amplitude of EEG, sometimes by an order of magnitude. Additionally, fewer trials were conducted during each condition for the pilot dataset, which may impact training on architectures that generally demand a large number of training examples, such as CNNs.

Mean accuracy and F1-Score were lower for all the CNN-based classifiers when trained on the pilot experiment dataset, although this difference was not statistically significant (Mann-Whintey U—CNN: *p* = 0.113, CNN_shallow: *p* = 0.054, CNN_LSTM: *p* = 0.063). As previously mentioned, this may in part be explained by the lower number of training examples. Perhaps the most striking difference between datasets is the sharper performance degradation as the signal time window to be classified is moved earlier, which occurred for both the best performing classifiers (MLP and deep CNN). This also resulted in a considerable reduction of the DTA provided in both cases. While the time advantage provided by the system is promising, this difference highlights the need for ecologically valid protocols when attempting to develop EEG-based action anticipation systems.

A potential explanation for the decrease in DTA could be differences between the brain responses elicited during motor execution tasks, as opposed to motor imagery. While potentials arising during both conditions are similar, they present slightly different network dynamics (Kim et al., 2018), and both oscillatory (Höller et al., 2013) and MRCP responses (Shakeel et al., 2015) may differ significantly. In our case, the steeper drop-off during Motor Execution tasks suggests the classification models may have leveraged quicker, later brain responses. This finding has implications for downstream research on the topic, suggesting distinct action anticipation pipelines may have to be developed for Motor Imagery and Execution, so as to ensure early MRCPs are consistently captured under both conditions.

## 6 Conclusion

We demonstrated the viability of action anticipation from EEG signals, in a Human-Robot Interaction scenario, using only single session and condition trials to train Deep Learning models. Additionally, a new metric to characterize a system's action anticipation capabilities was proposed and implemented. Our findings were built upon a publicly available MI-BCI EEG dataset (Farabbi et al., 2022), as well as a pilot experiment, with a Motor Execution condition, both of which involved Human-Robot Interaction tasks. Overall, the action anticipation system presented satisfactory performance, and was capable of consistently detecting action anticipation EEG signals by several hundred milliseconds, in line with other state-of-the-art methodologies.

Further work on this topic will include testing under closed-loop neurofeedback to characterize performance more realistically, followed by a self-paced HRI experiment, during which the potential complementary role of eye tracking for action anticipation will also be explored.

## Data Availability

The raw data supporting the conclusions of this article will be made available by the authors, without undue reservation.
